# A survey of nuclear ribosomal internal transcribed spacer substitution rates across angiosperms: an approximate molecular clock with life history effects

**DOI:** 10.1186/1471-2148-6-36

**Published:** 2006-04-25

**Authors:** Kathleen M Kay, Justen B Whittall, Scott A Hodges

**Affiliations:** 1 Department of Plant Biology, Michigan State University, 166 Plant Biology Building, East Lansing, MI, 48824, USA; 2 Department of Ecology, Evolution and Marine Biology, University of California, Santa Barbara, CA, 93106, USA; 3 Section of Evolution and Ecology, University of California, One Shields Ave., Davis, CA, 95616, USA

## Abstract

**Background:**

A full understanding of the patterns and processes of biological diversification requires the dating of evolutionary events, yet the fossil record is inadequate for most lineages under study. Alternatively, a molecular clock approach, in which DNA or amino acid substitution rates are calibrated with fossils or geological/climatic events, can provide indirect estimates of clade ages and diversification rates. The utility of this approach depends on the rate constancy of molecular evolution at a genetic locus across time and across lineages. Although the nuclear ribosomal internal transcribed spacer region (nrITS) is increasingly being used to infer clade ages in plants, little is known about the sources or magnitude of variation in its substitution rate. Here, we systematically review the literature to assess substitution rate variation in nrITS among angiosperms, and we evaluate possible correlates of the variation.

**Results:**

We summarize 28 independently calibrated nrITS substitution rates ranging from 0.38 × 10^-9 ^to 8.34 × 10^-9 ^substitutions/site/yr. We find that herbaceous lineages have substitution rates almost twice as high as woody plants, on average. We do not find any among-lineage phylogenetic constraint to the rates, or any effect of the type of calibration used. Within life history categories, both the magnitude of the rates and the variance among rates tend to decrease with calibration age.

**Conclusion:**

Angiosperm nrITS substitution rates vary by approximately an order of magnitude, and some of this variation can be attributed to life history categories. We make cautious recommendations for the use of nrITS as an approximate plant molecular clock, including an outline of more appropriate phylogenetic methodology and caveats against over interpretation of results. We also suggest that for lineages with independent calibrations, much of the variation in nrITS substitution rates may come from uncertainty in calibration date estimates, highlighting the importance of accurate and/or multiple calibration dates.

## Background

Decades after the idea of a molecular clock was first broached[1], the weight of evidence suggests that rate heterogeneity, not rate constancy, is the norm in molecular evolution (reviewed in [2,3]) and that an exact molecular clock will not be found. Nevertheless, an approximate clock can be established for a locus with many independent calibrations [4], and would be useful for roughly dating lineages and testing time-dependent evolutionary hypotheses. Local rate-constancy and even a single calibration can allow dating within a particular lineage, but much of the excitement surrounding molecular clocks stems from the possibility of approximating dates for the majority of lineages that lack independent calibrations. This has become commonplace in the animal kingdom, especially with the widely applied mtDNA clock [5,6], but a general molecular clock able to date relatively recent divergences in plants remains elusive. Because there will be variation among independently calibrated substitution rates, a first step in determining whether a particular locus may be useful as a molecular clock is to ascertain the amount and sources of this rate variation.

In the last decade, the nuclear ribosomal internal transcribed spacer region (nrITS) has revolutionized species-level plant phylogenetics. Because concerted evolution has generally homogenized sequence variation among the numerous ribosomal DNA copies within an individual, direct sequencing of this region is possible for most systems. This, coupled with the availability of universal primers and elevated substitution rates compared to most chloroplast regions, make it especially accessible and appropriate for resolving interspecific phylogenetic relationships [7,8]. Although reliance on nrITS as the sole source of phylogenetic evidence has come under criticism because of certain features of its evolution [9], it remains the most efficient locus for generating species-level phylogenetic inferences in most plant groups.

The widespread use of the nrITS region at the species level makes it a good candidate for a potential plant molecular clock. Substitution rates have been independently calibrated for a number of plant groups with diverse life histories and growth forms that represent distinct branches of the angiosperm phylogeny. Preliminary surveys of some of these rates suggest that they vary by almost an order of magnitude [10,11]. However, these limited surveys do not fully evaluate the variation in available nrITS substitution rates nor do they examine possible sources of this variation. Even without a full summary of nrITS rate variation, there is a growing trend in the literature to utilize some published rates for groups that lack their own independent calibration, either by choosing a rate based on life history similarity or phylogenetic relatedness, or by using a range of the more commonly-cited rates, such as Suh (1993) or Sang (1994, 1995). Here, we conduct a systematic search of the literature in order to identify and evaluate more thoroughly nrITS rate variation. Specifically, we report a summary of independently calibrated rates, examine potential correlates that may account for some of the variation in rates, and make conservative recommendations regarding the use of nrITS as an approximate molecular clock for angiosperms.

## Results

### Literature survey

**Figure 1:**
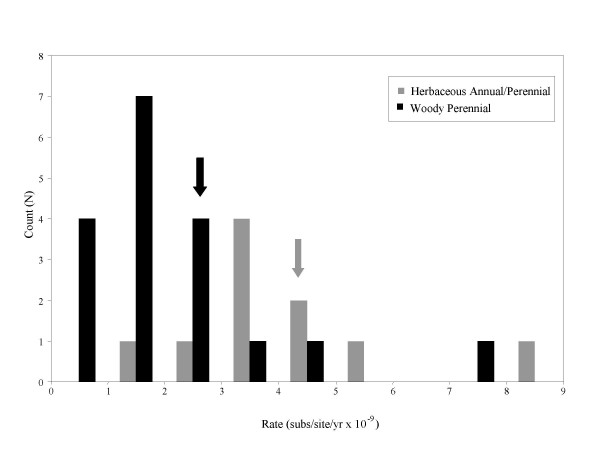
**The distribution of ITS rates**. A frequency histogram of nrITS rates, showing the difference between woody perennial and herbaceous annual/perennial rates. Arrows indicate the average rates for each life history category. The outlier rate for *Gentiana *Sect. *Cimialis *(19 × 10^-9 ^subs/site/yr) has been removed.

We identified 29 independent nrITS substitution rates ranging from 0.38 × 10^-9 ^subs/site/yr in *Hamamelis *to 19 × 10^-9 ^subs/site/yr in *Gentiana *Sect. *Ciminalis *(Fig. 1; Table 1). The latter rate was excluded as an outlier from all of our analyses since the calibration was based on only a single base-pair substitution and the rate was greater than double the second highest rate of 8.34 × 10^-9 ^subs/site/year in *Soldanella*. The mean of the 28 remaining rates was 2.86 × 10^-9 ^subs/site/yr. Of those studies reporting results of rate constancy tests, 12 out of 19 passed or partially passed (Table 1).

**Table 1:** Independently calibrated nrITS substitution rates sorted by magnitude of rate.

Taxon	Family	Life History^a^	Clock Test	Calibration Type^b^	Calibration Date (ma)	Rate^d^	Reference
*Hamamelis*	Hamamelidaceae	W	NA	G	8.5	0.38	[48]
Winteraceae	Winteraceae	W	NA	G	65^c^	0.45^e^	[27]
*Nothofagus*	Nothofagaceae	W	NA	F	83	0.50	[49]
Salicaceae	Salicaceae	W	NA	F	50^c^	0.60	[50]
*Aralia s*ect. *Dimorphanthus*	Araliaceae	W	NA	G	12	1.07	[51]
*Echium*	Boraginaceae	W	Passed	G	20	1.10	[52]
*Alnus*	Betulaceae	W	Rejected	F	70	1.10	[53]
Empetraceae	Empetraceae	W	Passed	F	37	1.44	[54]
*Saxifraga*	Saxifragaceae	H	NA	NA	5.5^c^	1.72	[55]
*Aesculus*	Hippocastanaceae	W	Passed	F	65	1.72	[56]
*Gaertnera*	Rubiaceae	W	Passed	F	54	1.99	[11]
*Ormocarpum*	Fabaceae	W	Rejected	G	35	2.00	[57]
*Inga*	Fabaceae	W	Rejected	G	3.5	2.34	[10]
*Phylica*	Rhamnaceae	W	Rejected	G	2	2.44	[58]
*Adansonia*	Bombacaceae	W	Passed	F	47^c^	2.48	[59]
*Eupatorium*	Asteraceae	H	Passed	M (*ndh*F)	14.8	2.51	[60]
Tarweeds/Hawaiian silverswords	Asteraceae	W	Passed	C	15	3.00	[61]
Robinioid legumes	Fabaceae	W	Rejected	F	39.4^c^	3.30	[62]
*Lupinus*	Fabaceae	H	NA	F	60	3.46^e^	[63]
*Astragalus*	Fabaceae	H	NA	F	35	3.50	[64]
Cucurbitoideae	Cucurbitaceae	H	NA	F	40	3.62	[65]
*Ehrharta*	Poaceae	H	Rejected	M (*rbc*L) & F	41	3.81	[66]
*Plantago*	Plantaginaceae	H	Rejected	G	0.6	4.27	[71]
*Gentianella*	Gentianaceae	H	Passed	G & F	3	4.52	[67]
*Dendroseris*	Asteraceae	W	Passed	G & M (cpDNA)	3.3^c^	5.00	[28]
*Gossypium*	Malvaceae	H	NA	F & M (cpDNA)	8.5^c^	5.50^e^	[68]
*Robinsonia*	Asteraceae	W	Passed	G	4	7.83	[69]
*Soldanella*	Primulaceae	H	Passed	F	23.3	8.34	[70]
*Gentiana *sect. *Ciminalis*	Gentianaceae	H	Passed	G	0.1	19.00	[25]

^a^W = woody, H = herbaceous.

^b^G = geographic vicariance; F = fossil; M = molecular clock from a different locus, followed by an identifier for that locus in parentheses; C = climatic.

^c^Calibration age and associated substitution rate are averages of two reported ages.

^d^substitutions/site/year × 10^-9^.

^e^Substitution rate is a weighted average of ITS1 and ITS2 rates reported separately.

### Phylogenetic signal

Twenty-one different angiosperm families are represented among the 29 rates, which comprise non-overlapping portions of the Angiosperm evolutionary tree. No significant phylogenetic signal to the rates was detected (p = 0.393), suggesting that phylogenetic relatedness is not an appropriate justification when choosing rates from the literature. Indeed, rates from the family Asteraceae alone span almost the entire range found in Table 1, from 2.5 × 10^-9 ^subs/site/year in *Eupatorium *to 7.83 × 10^-9 ^subs/site/year in *Robinsonia*.

### Life history effects

The average nrITS substitution rates for herbaceous and woody lineages are significantly different (p = 0.0013, Wilcoxon Rank Sum Test; Fig. 1). The herbaceous annual/perennial rates ranged from 1.72 × 10^-9 ^to 8.34 × 10^-9 ^substitutions/site/year (mean = 4.13 × 10^-9 ^subs/site/yr, N = 10), and the woody perennial rates ranged from 0.38 × 10^-9 ^to 7.83 × 10^-9 ^substitutions/site/year (mean = 2.15 × 10^-9 ^subs/site/yr, N = 18). In our analysis of phylogenetic independent contrasts, we found that nine of ten life history contrasts exhibit a lower rate associated with the woody perennial lineage (Sign Test, p < 0.01). The single opposing contrast involved the Hawaiian silverswords. This rate is based on a clade that includes both the woody perennial silverswords and the herbaceous perennial tarweeds. Although initially coded as woody perennials, this state is considered the derived condition among the tarweeds. Therefore, the rate may be more representative of the herbaceous perennial ancestry in the clade than the derived woody perennial silversword lineage. Our contrast results are robust to either life history classification of the tarweed/silversword rate (alternative coding, p < 0.01).

## Discussion

### Range of nrITS substitution rates

This study represents the most comprehensive collection of nrITS substitution rates to date. The largest previous survey [10] included independently calibrated rates from 12 angiosperm lineages. They recommended using a range of rates from 1.72 × 10^-9 ^to 7.83 × 10^-9 ^for dating the diversification of *Inga*, after removing the rate from Winteraceae because of its longer generation time and possible error associated with the calibration date. The addition of 16 independently calibrated rates in our study introduces seven new rates slower than 1.72 × 10^-9^, one of which is slower than the Winteraceae rate. Our broader sampling of nrITS substitution rates allowed us to analyze more fully potential sources of variation in these rates.

### Life history effects

Striking differences in rates of nrITS evolution between life history categories, with annuals/herbaceous perennials exhibiting significantly higher rates, are consistent with a general pattern of higher substitution rates for annual compared to perennial plants and for herbs compared to woody trees that has been reported from chloroplast [12-16] and mitochondrial studies [17]. Life history has also been suggested to affect rates specifically of nrITS substitution ([18,19] but see [20]), with annual species exhibiting longer nrITS branch lengths than perennials. In a more comprehensive review, however, Whittle and Johnston [21] found no difference in nrITS branch lengths between sister taxa differing in annual versus perennial life history. Our study may be more likely to detect rate differences associated with life history because we are using more distinctly polarized life history categories and our comparisons are across deeper phylogenetic splits, with more time for rate differences to accumulate.

Although the underlying mechanism is unclear, our results agree with previous studies in which shorter generation times are associated with faster rates of molecular evolution [22,23]. It is difficult to explain how generation time would affect substitution rates in plants, which do not sequester their germ lines and therefore can pass on somatic mutations to their gametes, unless the majority of inherited mutations arise during meiosis and not mitosis. Furthermore, we do not know whether generation time per se is a causative factor of substitution rate variation. Differences in generation time in plants are confounded by correlated differences in growth form [21] and mating system [24], such that perennials are the only plants forming extensive wood and annuals are much more frequently self-fertilizing. A mechanistic understanding of the effects of these factors on rates of molecular evolution will increase the power of molecular clock approaches in plant evolution.

### Errors in rate estimation

A potentially confounding factor associated with our life history classes is the type of calibration point used and its potential associated rate biases. Fossil data typically provide a minimum age of a particular lineage, and therefore may be more likely to *overestimate *a substitution rate, while biogeographic or climatic events provide a maximum age and may be more likely to *underestimate *a rate. Thus, an unacknowledged correlation between life history and calibration type potentially could confound our results for rate differences between woody and herbaceous plants. These potential biases appear not to be an issue. First, our dataset is well balanced with fossil calibrated rates for eight woody perennial and for six herbaceous annual/perennial groups. There is no overall correlation between type of calibration and life history category (χ^2 ^= 0.156, ns). Second, within each life history category, we see no apparent bias due to the type of calibration. For example, within the herbaceous rates, only one of the five highest rates is calibrated exclusively with fossil evidence. Similarly, of the five slowest woody perennial rates, three are biogeographic and two are fossil calibrated.

**Figure 2:**
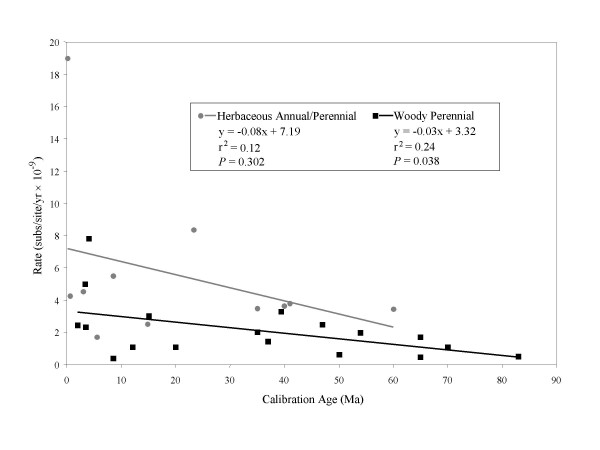
**The effects of calibration age**. A comparison of nrITS substitution rates with calibration age reveals higher variance at younger calibration points. The decreasing substitution rate with older calibration ages is only significant for the woody perennials. The herbaceous annual/perennial regression is not significant and becomes even less so when the outlier is removed (p = 0.861).

The age of the calibration point may affect the precision of the substitution rate estimates, since more recent calibration ages are based on fewer "ticks" of the molecular clock. Older lineages will have more nucleotide substitutions and thus these rate estimates should become more precise, at least until they become biased by sequence saturation, or multiple substitutions at the same site. Among our 29 original rates, calibration ages ranged from 0.1 Ma to 83 Ma. There appears to be substantially more variation in substitution rates based on recent calibration points compared to those from older calibration ages in both life history categories (Fig. 2). An extreme example is the exceptionally high rate from *Gentiana *sect. *Ciminalis*, that was more than twice as high as any other rate. This rate was calibrated based on a biogeographic split of only 0.1 Ma after which only one nucleotide substitution occurred [25]. When rate is regressed on age of calibration, separately for each life history category, the residuals exhibit a negative trend with increasing calibration age indicating lower variance for older calibration ages (Fig. 2). Another indication of this trend is found by comparing the variation in residuals for rates with calibration points older and younger than 30 Ma. The mean absolute value of these residuals in the more recently calibrated half is more than three times higher than in the half with older calibrations.

We also found a trend of decreasing rate with the age of calibration, which was significant among woody perennials (Fig. 2). This trend may be due to the models of sequence evolution utilized for estimating sequence divergence. Without correcting for sequence saturation, rate estimates are expected to decrease with increasing time since divergence [5,6]. In molecular clock calculations, using a model of sequence evolution that does not fully capture sequence saturation and among-site rate variation potentially can lead to large errors in divergence time estimates (reviewed in [3]). Most of the original rates we found used the relatively simple Jukes-Cantor or Kimura 2-parameter models, so this might be a significant issue, particularly for studies with older calibration dates. Ideally, objective criteria for model selection, such as the likelihood ratio tests easily implemented in MODELTEST [26] should be used before calculating a rate.

As a post hoc exploration of the effects of methods of estimating sequence divergence, including the choice of substitution model and the treatment of rate heterogeneity among branches, we reanalyzed the sequence data used to estimate two of the nrITS rates, for the Winteraceae [27] with one of the oldest calibration dates and for *Dendroseris *[28] with a recent calibration. These are also two of the most frequently cited rates in the literature. For the Winteraceae, our resulting substitution rate estimates, based on a TrN+G substitution model and penalized likelihood rate smoothing, are higher than crude estimates based on uncorrected counts of pairwise differences. However, the uncertainty reported for their biogeographic calibration – the isolation of Pseudowinteraceae in Australia – for which estimates range from 50 to 80 Ma, affected the overall rate estimates substantially more than the different methods of estimating branch lengths (substitutions/site/year × 10^-9^: 50 Ma calibration crude = 0.52, corrected = 0.65; 80 Ma calibration crude = 0.33, corrected = 0.41). Importantly, for this group with one of the oldest calibrations, which should be more sensitive to the effects of sequence saturation and rate heterogeneity, branch length estimation methods make a small difference in light of the overall range of nrITS substitution rates in our survey, not even affecting its category in the histogram of Figure 1. For *Dendroseris*, there was no significant rate heterogeneity, and using a more complex substitution model had no effect on the substitution rate estimate. In contrast, there was substantial variation in rates due to uncertainty in the biogeographic calibration, ranging from 4 Ma based on the age of Masatierra Island to 2.6 Ma based on a chloroplast DNA clock calculation (substitutions/site/year × 10^-9^: 4 Ma calibration = 3.94; 2.6 Ma calibration = 6.06). These reanalyses suggest that fine-tuning the estimates of molecular substitution makes a relatively small change to the overall rate estimate in light of substantial uncertainty in calibration dates. While branch length estimates clearly should be optimized, there is also a real need for more accurate and multiple calibrations to reduce the uncertainty of rate estimates [29].

### An approximate plant molecular clock?

The variance we find in nrITS substitution rates is likely the result of both error in individual rate estimates and biological differences among lineages in actual substitution rates. As noted above, substantial error in estimating individual rates can come from uncertainty in the dating of the fossil or biogeographic/climatic event used as a calibration and in the assignment of that calibration to a node in the phylogeny [29,30]. Further error is contributed by uncertainty in the phylogeny itself, both in the branching topology and the branch length estimates [31]. Another issue is that gene trees are not identical to species trees [3]. Coalescence of nrITS alleles should predate species divergences by a factor related to the effective population size, and any reticulate evolution or lineage sorting could obscure the true tree topology [32]. In attempting to date a particular plant lineage, few studies adequately consider these sources of error.

While some of the variance in the rates we found likely comes from these sources of error, there are also very real differences in nrITS substitution rates within and among lineages. Statistically significant rate heterogeneity was found in seven of the nineteen studies reporting tests of rate constancy. Furthermore, in spite of all the sources of noise in our dataset, we found a substantial effect of life history, with long-lived woody plants exhibiting much slower substitution rates than shorter-lived herbaceous lineages. Thus even if error in substitution rate estimates could be eliminated, the existence of a universal nrITS substitution rate is highly unlikely.

Despite the many sources of error in rate estimates, however, we find a limited distribution of rates with a central tendency, especially when separated by life history category (Fig. 1). We therefore support the cautious use of this locus as a general molecular clock for roughly dating events in plant evolution, but we offer a few important caveats. Borrowing just one or a few rates from the literature, especially based on phylogenetic relatedness, does not adequately account for the nature and extent of variation in the nrITS "clock". Instead, to roughly estimate a date we suggest that, within a life history category, the entire range of rates be considered. Furthermore, before using any rate for dating historical events, whether calculated from an internal calibration or borrowed from the literature, nrITS rate constancy should be tested within the group of interest, ideally with a tree-based likelihood ratio test [33,34], and any lack of local rate constancy should be considered in the dating methodology [35].

The distribution of rates could then be used to examine specific hypotheses for the timing of events for lineages without calibrations of their own. For example, if there is a hypothesis for the age of a particular node in the phylogeny of a group of taxa with an nrITS dataset, one could calculate the required substitution rate to fit that age to the node and then compare the required rate to the distribution of rates presented here. A good example of this type of hypothesis testing is found in the genus *Aquilegia *(Ranunculaceae). Hummingbird pollination in this group was suggested to have originated during the mid-Pliocene (ca. 3.5 Ma) based on floristic associations [36]. Hodges et al. [37] calculated the nrITS substitution rate necessary within *Aquilegia *to fit the hypothesized age of the hummingbird pollination syndrome (0.11% per my = 0.56 × 10^-9 ^subs/site/yr). This is approximately ten times slower than the average annual/perennial herbaceous rate (4.13 × 10^-9^) and more than three times slower than the slowest annual/perennial herbaceous value. Even using the slowest annual/perennial rate (1.72 × 10^-9^), the estimated age of hummingbird pollination is only half as old as predicted (approximately 1.66 Ma). Without any justification for such an unusually slow nrITS substitution rate in *Aquilegia*, hummingbird pollination likely has evolved more recently than originally predicted [37].

## Conclusion

The results from our literature survey underscore the popularity of the nrITS region as a tool to date relatively recent historical events in plant evolution. The original rates that we found came from a broad taxonomic spectrum representing a diversity of life histories and were calculated using a variety of calibrations and methodologies. Nevertheless, the rates were consistent to approximately an order of magnitude, or even less for herbaceous lineages considered separately. Although this survey should be considered an ongoing project, given these initial results, the nrITS may be useful as an approximate molecular clock for roughly dating divergences, calculating diversification rates, and hypothesis testing in plant groups that currently lack a fossil or biogeographic calibration of their own.

## Methods

### Literature survey

We surveyed *Systematic Botany*, *Molecular Phylogenetics and Evolution*, *The American Journal of Botany*, *Evolution *and any references therein, from January 1995 through December 2003. We recorded rates for any study that independently calibrated and reported a rate for ITS1 and ITS2. If multiple rates were reported, we followed the author's recommendation on which was likely to be most accurate, unless their reasoning was based on the rate's similarity to other published rates. In such cases, we took the average of the reported rates. When separate rates were reported for ITS1 and ITS2, a weighted average based on the average length of each nrITS region was calculated. For each rate, we also recorded the predominant growth form/life history [38,39], rate constancy test results, calibration type, and calibration age.

### Phylogenetic signal

Frequently, authors that utilize published rates in their own studies base their choices on phylogenetic relatedness. In order to determine whether such choices are warranted, we tested for any phylogenetic signal in the rates. First we constructed a phylogeny of the taxa represented in our rates survey using the web-based program Phylomatic [40] and then manually edited the tree to reflect recent and more detailed phylogenetic hypotheses for some families [41-43] (tree available upon request). The Test for Serial Independence was implemented in the program Phylogenetic Independence v. 2.0 [44,45] with 1000 randomizations. This test compares the autocorrelation of adjacent branches of the original tree to that of a series of randomized trees.

### Life history effects

Generation time has been suggested to affect rates of molecular evolution [22,23], and thus we classified each rate according to the predominant life history of the clade, either annual/herbaceous perennial or woody perennial, as a proxy for generation time. The herbaceous category consists of relatively short-lived plants that reach sexual maturity within one to a few years, while the woody perennial life history category consists of long-lived lineages. It is possible that phylogenetic relatedness and life history could be confounded. Thus, to be conservative we also tested for rate differences due to life history using phylogenetically independent contrasts. Contrasts were identified with the BRUNCH algorithm in CAIC [46], using the same cladogram as in the above mentioned Test for Serial Independence. BRUNCH locates nodes for which there is a contrast in life history, and then calculates the direction of the rate difference across that node, using each taxon in no more than one contrast. It assumes that if sister taxa have the same life history state their most recent common ancestor also had that state, and that, when branch lengths are unknown, the rate for this ancestor can be calculated as the average rate of the derived taxa.

### Reanalysis of Winteraceae and *Dendroseris *examples

We reanalyzed the sequence data used to estimate the nrITS rates for the Winteraceae (GenBank accessions AY004111-AY004128; [27] Additional File 1) and for *Dendroseris *[28] Additional File 2. We aligned the sequences with ClustalX and refined the alignments manually. Phylogenetic relationships were estimated in PAUP 4.0b10 [47] using maximum likelihood and a TrN+G substitution model for Winteraceae and a TrNef model for *Dendroseris*, as chosen by MODELTEST. For Winteraceae, we found significant substitution rate heterogeneity in nrITS using a tree-wide likelihood ratio test for differences between clock-constrained and clock-unconstrained trees (p < 0.01; [34]). We then used penalized likelihood rate smoothing implemented in R8S [35] to estimate branch lengths and substitution rates in the face of this heterogeneity. Within *Dendroseris*, we did not find significant rate heterogeneity with a tree-wide LRT (p > 0.1), and therefore rate smoothing was unnecessary for estimating substitution rates.

## Authors' contributions

JW and KK designed and coordinated the study, carried out the literature survey, performed the analyses, and drafted the manuscript. SH provided original direction and helped to revise the manuscript. All authors discussed and interpreted the results and read and approved the final manuscript.

## Supplementary Material

Additional File 1 This is the alignment of Winteraceae nrITS from Genbank accessions AY004111-AY004128[27], used in calculating the effects of calibration uncertainty and branch length estimation on substitution rate. It includes PAUP and r8s command blocks and the tree description from the maximum likelihood analysis. Click here for file

Additional File 2 This is the alignment of Dendroseris nrITS from Sang [28] used in calculating the effects of calibration uncertainty and branch length estimation on nrITS substitution rate. It includes a PAUP command block and the tree description from the maximum likelihood analysis. Click here for file
